# The impact of functional excess of footwear on the foot shape of 7-year-old girls and boys

**DOI:** 10.7717/peerj.11277

**Published:** 2021-04-20

**Authors:** Ewa Puszczalowska-Lizis, Aleksandra Lukasiewicz, Sabina Lizis, Jaroslaw Omorczyk

**Affiliations:** 1 Medical College, Institute of Health Sciences, University of Rzeszow, Rzeszow, Poland; 2 Non-Public Physiotherapy Practice, Strzyzow, Poland; 3 Faculty of Physical Education and Sport, Institute of Sport, University of Physical Education in Krakow, Krakow, Poland

**Keywords:** Public health, Human foot, Footwear fit, Indoor shoes, Functional length excess, Functional width excess, Feet deformities, Regression analysis, Health policy, Children

## Abstract

**Background:**

Properly fitted shoes can support the development of growing feet and prevent problems and pathologies, not only in childhood, but also in adulthood. This points to the need to conduct research on the impact of shoe fitting on the structure of the foot in order to raise awareness and importance of this problem, to enable proper decisions regarding the purchase and use of shoes. The aim of this study was to analyze indoor footwear fit and its impact on foot structure in 7-year-old schoolchildren.

**Methods:**

The CQ-ST podoscope and the Clevermess device were used for measurements. The analysis was carried out using the Mann Whitney U test, Wilcoxon signed-rank test, Chi-square test, regression analysis.

**Results:**

About 40% of girls and boys had shoes that were incorrectly fitted in length, while as many as 74% of girls and 66% of boys wore shoes that were incorrectly fitted in width. Regression analysis demonstrated a statistically significant influence of the footwear length on longitudinal arch of the right and left foot and the transverse arch of the right foot. In boys, the length of the shoes shows associations with the right and left hallux valgus angle.

**Conclusion:**

A significant percentage of the studied 7-year-olds, regardless of gender, wears inappropriately fitted shoes. In both sexes, the length of the footwear influenced the longitudinal arch of the right and left foot and the transverse arch of the right foot. Due to the deformity of the first metatarsophalangeal joint, the boys with hallux valgus require footwear which is wider and therefore their shoes need to be bigger in size.

## Introduction

The human foot is a supporting and load-carring part of the musculoskeletal system and is shaped individually for each person. Numerous complex functions depend on the correct structure and operation of its individual components. The reasons that largely influence this part of the musculoskeletal system include genetic, environmental, socio-economic, lifestyle and the type of footwear worn (Jiménez-Ormeño et al., 2011; Jiménez-Ormeño et al., 2013; Watanabe et al., 2014; Dinato et al., 2015; Kinz, Groll-Knapp & Klein, 2015; Barisch-Fritz, Plank & Grau, 2016; Hettigama, Punchihewa & Heenkenda, 2016; Tong & Kong, 2016; Tay et al., 2017; Puszczalowska-Lizis et al., 2019).

Children’s feet are particularly sensitive to external factors as they are undergoing developmental processes (Medina-Alcántara et al., 2019). From the developmental point of view, the early school period is one of the most important stages. This period is characterized by a moderate increase in the length characteristics of the body, which creates favorable static and dynamic conditions, however, due to the change in a lifestyle caused by beginning of school education, it is considered to be critical for posturogenesis (Kellis & Emmanouilidou, 2010). Children shift from a free, largely individually regulated daily rhythm to sitting for many hours a day. The type of footwear used to move around the school building is also changing. Soft slippers, previously worn in kindergarten, are replaced with indoor footwear. Most parents choose shoes for their children made of light, airy materials, with a rubber, non-slippery sole (Ma, Ai & Meng, 2012; Yurt, Sener & Yakut, 2014; Puszczalowska-Lizis et al., 2020).

The correct selection of footwear is of particular importance for children, mainly due to the dynamics of the development of the musculoskeletal system and the loads to which the lower parts of the kinematic chain are subjected to. However, this choice is often more influenced by aesthetic, economic or marketing considerations than by health considerations (Medina-Alcántara et al., 2019). Parents of girls especially try to ensure that the shoes, apart from being practical, have a fashionable and attractive appearance (Zhang & Wang, 2017). Wang, Chen & Lin (2014) emphasized that in response to aesthetic standards and parents’ requirements, shoes with a design stylized as shoes for adults, e.g., with narrow toes, are produced. Other studies show that in response to rising prices, parents decide to buy shoes for the so-called “over-the-top”, i.e., longer than the length of the children’s’ feet. Too much excess room in the footwear may cause the dimensions of the shoe to be inconsistent with the dimensions of individual parts of the foot, and therefore lead to the foot moving, the feeling of instability and discomfort, and even cause falls (Knapik, 2000; Medina-Alcántara et al., 2019). On the other hand, parents rarely monitor the length and width features of the feet and the current fit of the footwear (Yurt, Sener & Yakut, 2014; Kinz, Groll-Knapp & Klein, 2015; Zhang, 2016). The fit, understood as the matching of the foot shape with the inner space of the shoe, is optimal when the individual parts of the foot correspond to the individual parts of the footwear (Pavlackova et al., 2015).

Properly fitted shoes can support the development of growing feet and prevent problems and pathologies, not only in childhood, but also in adulthood. This points to the need to conduct research on the impact of shoe fitting on the structure of the foot in order to raise awareness and importance of this problem, to enable proper decisions regarding the purchase and use of shoes. This was the reason for undertaking the subject of the paper, the aim of which is to analyze indoor footwear fit and its impact on foot structure in 7-year-old girls and boys.

To this end the following research questions were meant to be addressed:

1. Are the right and left feet of the examined children characterized by symmetry in terms of length, width, longitudinal arch, transverse arch, position of the hallux and the Vth toe?
2. What is the frequency of wearing improperly fitted shoes and is it dependent on gender?
3. Does the size of the functional excess of the footwear length and width differentiate the feet of the examined girls and boys?
4. Are the length and width of the footwear worn by the examined girls and boys related to the features of their feet?

## Material & Methods

### Participants

We examined 7-year-old children (50 girls, 50 boys) attending the randomly selected schools in the Strzyzow administrative district. The representative size of the sample was calculated taking into account 95% confidence level, as well as 5% of admissible error for fraction estimation. The total number of children amounted to 139.

The following inclusion criteria were applied: age 7 years; informed consent provided by parent or legal guardian; lack of any diseases, congenital malformations, injuries of the musculoskeletal system, previous surgical procedures performed on the foot. We excluded children who didn’t understand the instructions that were necessary for the measurement procedures and/or refused to participate in the study.

After ending of the allocation procedure, it was established that 39 children couldn’t take part in the study protocol due to their non-compliance with the selection criteria. Out of the remaining candidates, 2 equal-sized, 50-person groups were made, in view of the sex.

Anthropometric measurements of the body mass and height were taken. Body weight measurements were made using a SECA 635 medical scales *(Seca, Germany)*. The results were read with an accuracy of 0.1 kg. Body height was measured in a standing position using a SECA 264 stadiometer (*Seca*, * Germany*), with an accuracy of 0.1 cm. The head was set in the natural, upright position, with eyes looking straight at a mark. The upper limbs along the body, the feet hip-width apart. In such a position, the body height was the distance of the highest point of the head from the base on which the examined child was standing. The obtained data were used to calculate the Body Mass Index (BMI). The basic descriptive statistics of the bodily characteristics of the examined children are presented in Table 1.

**Table 1:** Comparison of select bodily characteristics in respective groups of subjects.

Variable	$\overline{x}$ ± SD	Me	*Z*_1_	*p*
Body mass [kg]				
Girls	26.65 ± 2.97	26.25	−1.68	0.094
Boys	27.95 ± 3.87	27.25		
Body height [cm]				
Girls	125.32 ± 4.69	126.00	0.23	0.817
Boys	125.34 ± 4.27	125.50		
BMI				
Girls	16.94 ± 1.34	16.87	−1.76	0.078
Boys	17.78 ± 2.08	17.57		

**Notes.**

x arithmetical mean value SD standard deviation Me median *Z*_1_ value of the Mann Whitney U test statistic *p* probability value

### Procedures

The study protocol entailed the feet measurement standing in a relaxed position, with the arms along the body, and body weight distributed evenly on both feet. The CQ-ST podoscope (*Electronic System, Poland*) was applied for the evaluation of foot structure. The following indices were measured (Klein et al., 2009; Pita-Fernández et al., 2015; Puszczalowska-Lizis et al., 2020):

−  foot length –the line connecting the most distal point of the forefoot with the farthest point within the hindfoot, in cm;

– foot width –the line connecting the most medially located point on the head of the Ist metatarsal bone (*metatarsale tibiale, mtt*) with the point lying most laterally on the head of the Vth metatarsal bone (*metatarsale fibulare, mtf*), in cm;
– Clarke’s angle (longitudinal arch of the foot) –the angle between the tangent to the medial edge of the foot and the line joining the point of the largest recess of the footprint with the mtt point, in ^∘^;
– heel angle (*γ*) (transverse arch of the foot) –the angle between the tangents to the lateral and medial edge of the foot, which cross over the heel, in ^∘^;
– hallux valgus angle (*α*) –the angle between the tangent to the medial edge of the foot and the tangent to the pad of the Ist toe, derived from the *mtt* point, in ^∘^;
– the angle of the varus deformity of the Vth toe (*β*) –the angle between the tangent to the lateral edge of the foot and the tangent to the pad of the Vth toe, derived from the *mtf* point, in ^∘^.

The footwear worn by the children inside the school (indoor footwear) were examined. The indoor shoes usually worn by schoolchildren in Poland include sneakers made of canvas with laces, a toe which is rounded, stiffened and made of rubber with stiffened heel, the sole is a flat and made of rubber preventing slipping and ensuring stability. The use of the tested footwear in girls ranged from 0.50 to 3.50 months ($\overline{x}=1.60 \pm  0.73$ months), and in boys ranged from 0.50 to 3.50 months ($\overline{x}=1.59 \pm  0.75$ months). Footwear fit was examined using the Clevermess device (*Clevermess, Germany*). The reliability of this measuring tool is excellent: ICC = 0.993 for length and ICC = 0.992 for width (Puszczalowska-Lizis et al., 2020). The following ranges were included when assessing footwear fitting in terms of length: the functional length excess less than 8 mm - too short footwear; the functional length excess from 8 to 12 mm - appropriate footwear; the functional length excess over 12 mm - too long footwear (Knapik, 2000; Rajchel-Chyla et al., 2012; Puszczalowska-Lizis et al., 2020). In terms of width the following ranges were involved: the functional width excess less than 1 mm - too narrow footwear; the functional width excess from 1 to 3 mm - appropriate footwear; the functional width excess over three mm - too wide footwear when (Knapik, 2000; Puszczalowska-Lizis et al., 2020).

The perception of footwear fit was evaluated subjectively using the modified 100 mm Visual Analog Scale (VAS), with face drawings on extreme poles and additionally verbal determination of the comfort of the footwear. The face labelled on the left end was contorted with a grimace which corresponded to the term “poorest fit possible” and on the right end was placed a smiling face which corresponded to the term “best fit possible”. The children were instructed on how to mark the answers to the question about footwear comfort on the scale. The results on the scale were marked in the presence of the examiner, while child wearing indoor shoes.

The authors performed all measurements in a gym, in the morning, using the same measurement tools to ensure integrity of the research process. The subjects didn’t perform intense physical activity 12 h prior to the measurements. Children were wearing their gymnastic uniforms. For the podoscopic examination, they took off their shoes and socks, while footwear fit was measured with socks. The participants’ feet were disinfected and dried prior to measuring. All procedures were carried out in full compliance with the Declaration of Helsinki. Participants, their parents or legal guardians were previously informed about the study and gave their written informed consent to participate in the research. The study protocol was approved by the Bioethics Review Committee of the Rzeszow University (Approval Ref. No. 3/12/2015).

### Statistical analysis

The normalcy of distribution of particular characteristics was verified by means of the Shapiro–Wilk test. The assessment of intergender differences in the average level of the examined variables was made using the Mann–Whitney U test. Right and left foot results were compared using the Wilcoxon signed-rank test. The dependences between the frequency of wearing correctly and incorrectly fitted footwear and gender were analyzed using the Pearson Chi-square test. Regression analysis was used to predict the values of the length and width functional excess of indoor footwear (independent variables) on the features of the foot structure (dependent variables). The starting point was to check whether the residual distributions (differences between the observed value and the one calculated from the regression equation) are subject to normal distributed. The measure of the extent to which the models explained the dependent variables were the values of determination coefficients (R^2^). The coefficients describe the part of the explained variability which results from its dependence on the explanatory variables included in the model. The R^2^ coefficients were calculated by squaring the multiple correlation coefficient R, which is a measure of the correlation of all predictors taken together with the dependent variable. The coefficient R^2^ multiplied by 100% indicates how many percent of the dependent variable’s variance (its variability) is explained by the predictor. The prediction of the dependent variable was made on the basis of the coefficient b of slope of the regression line. The influence of predictor variables on the dependent variable was analyzed on the basis of partial correlations. Partial correlation coefficients (r_p_) inform how much of a given predictor variable affects the dependent variable, but only that part (variance) that has not been explained by other predictors.

The probability of making an error of the first type (family-wise type-I error) was set at the level of *α* = 0.05. All calculations were conducted using the program STATISTICA Stat Soft, version 13.1.

## Results

The comparison of the structural features of the right and left foot showed statistically significant differences in the values of the varus angle of the Vth toe (*β*). This angle for the right foot reached higher values in both girls (*p* < 0.001) and boys (*p* < 0.001). Moreover, in boys, statistically significantly higher values of the right foot heel angle were found: *p* = 0.038 (Table 2).

In girls, the values of the functional excess of length were statistically significantly lower than in boys, both in the case of right (*p* = 0.049) and left (*p* = 0.035) shoes. There were no statistically significant differences between the sexes in the values of the excess for width recorded for right and left shoes, although the values of standard deviations indicate a large differentiation of the values of the analyzed variables (Table 3).

Data in Table 4 indicated over 60% of girls and boys had correctly fitted shoes in terms of length. Too short right footwear was worn by 30% of girls and 24% of boys, while the left was worn by 32% of girls and 24% of boys. Too long left and right footwear was found in 2% of girls and 14% of boys.

**Table 2:** Comparison of the values of foot structure indices obtained for the right and left foot.

Variable	$\overline{x}$ ± SD	max–min	*Q*_25_	Me	*Q*_75_	*Z*_2_	*p*
Foot length [cm] –girls							
Right	18.66 ± 0.94	20.00–16.70	18.00	18.80	19.40	0.00	1.000
Left	18.66 ± 0.94	20.00–16.70	18.00	18.80	19.40		
Foot width [cm] –girls							
Right	6.96 ± 0.49	8.00–5.80	6.80	7.00	7.20	0.46	0.648
Left	6.95 ± 0.48	7.70–5.20	6.70	7.00	7.20		
Clarke’s angle [^∘^] –girls							
Right	42.10 ± 5.75	53.00–28.00	38.00	42.00	46.00	0.80	0.421
Left	42.48 ± 6.21	56.00–25.00	40.00	43.50	47.00		
Heel angle *γ* [^∘^] –girls							
Right	16.76 ± 1.94	21.00–12.00	16.00	17.00	18.00	0.80	0.423
Left	16.44 ± 2.26	21.00–11.00	15.00	16.50	18.00		
Hallux valgus angle *α* [^∘^] –girls							
Right	3.20 ± 3.92	14.00–0.00	0.00	1.00	7.00	1.05	0.290
Left	3.96 ± 4.47	16.00–0.00	0.00	2.00	7.00		
The angle of the varus deformity of the Vth toe *β* [^∘^] –girls							
Right	14.56 ± 6.88	32.00–0.00	11.00	15.50	18.00	3.74	<0.001^*^
Left	10.86 ± 5.83	22.00–0.00	8.00	11.00	15.00		
Foot length [cm] –boys							
Right	19.29 ± 1.01	21.70–17.20	18.50	19.20	20.00	0.00	1.000
Left	19.29 ± 1.01	21.70–17.20	18.50	19.20	20.00		
Foot width [cm] –boys							
Right	7.20 ± 0.50	8.30–6.30	6.80	7.20	7.60	0.13	0.893
Left	7.21 ± 0.53	8.30–6.50	6.80	7.15	7.70		
Clarke’s angle [^∘^] –boys							
Right	36.18 ± 11.17	52.00–10.00	30.00	39.00	45.00	0.81	0.412
Left	37.26 ± 10.31	51.00–10.00	32.00	40.00	45.00		
Heel angle *γ* [^∘^] –boys							
Right	16.46 ± 2.22	21.00–12.00	15.00	16.00	18.00	2.06	0.038^*^
Left	15.80 ± 2.05	22.00–12.00	14.00	16.00	17.00		
Hallux valgus angle *α* [^∘^] –boys							
Right	4.82 ± 3.61	14.00–0.00	1.00	5.00	7.00	0.71	0.480
Left	5.42 ± 4.59	18.00–0.00	0.00	5.50	8.00		
The angle of the varus deformity of the Vth toe *β* [^∘^] –boys							
Right	15.28 ± 5.51	30.00–6.00	12.00	15.00	19.00	3.84	<0.001^*^
Left	10.84 ± 6.12	24.00–0.00	6.00	10.50	16.00		

**Notes.**

x arithmetical mean value SD standard deviation max maximum value min minimum value *Q*_25_ lower quartile Me median *Q*_75_ upper quartile *Z*_2_ value of the Wilcoxon signed-rank test statistic *p* probability value

* *α* < 0.05.

Correctly fitted footwear in terms of width was worn by 26% of girls and 34% of boys in the right foot, while the left was worn by 30% of girls and 32% of boys. Too narrow right footwear was worn too by 56% of girls and 50% of boys, while the left was worn by 42% of girls and 52% of boys. Too wide right footwear was found in 18% of girls and 16% of boys, while the left was worn by 26% of girls and 16% of boys (Table 4).

**Table 3:** Inter-gender differences in the functional length and width excess of footwear.

Variable	$\overline{x}$ ± SD	max–min	*Q*_25_	Me	*Q*_75_	*Z*_1_	*p*
Length excess [mm] –right shoe							
Girls	9.04 ± 2.18	13.00–5.00	7.00	8.00	11.00	−1.95	0.049^*^
Boys	10.04 ± 2.57	14.00–6.00	8.00	10.00	12.00		
Length excess [mm] –left shoe							
Girls	8.96 ± 2.18	13.00–5.00	7.00	8.00	11.00	−2.10	0.035^*^
Boys	10.04 ± 2.57	14.00–6.00	8.00	10.00	12.00		
Width excess [mm] –right shoe							
Girls	0.10 ± 3.14	9.00–(−6.00)	−2.00	−1.00	2.00	0.19	0.846
Boys	0.16 ± 3.86	12.00–(−6.00)	−3.00	0.00	3.00		
Width excess [mm] –left shoe							
Girls	0.92 ± 3.60	8.00–(−7.00)	−1.00	1.00	4.00	1.08	0.279
Boys	0.42 ± 3.69	12.00–(−5.00)	−2.00	−1.00	2.00		

**Notes.**

x arithmetical mean value SD standard deviation max maximum value min minimum value *Q*_25_ lower quartile Me median *Q*_75_ upper quartile *Z*_1_ value of the Mann-Whitney U test statistic *p* probability value

* *α* < 0.05.

No statistically significant dependencies were found between gender and footwear fit in relation to length and width (Table 4).

Participants reported very favourable perceptions of indoor footwear fit, with scores ranging from 83 to 100 mm in girls ($\overline{x}$_V AS_ = 93.79 ± 5.59 mm) and from 84 to 100 mm in boys ($\overline{x}$_V AS_ = 95.51 ± 5.12 mm).

**Table 4:** Passivity of indoor footwear depending on gender.

Passivity	Girls		Boys		Total	
	*n*	%	*n*	%	*n*	%
Fitting the footwear in terms of length –right foot						
Too short	15	30.0	12	24.0	27	27.0
Appropriate	34	68.0	31	62.0	65	65.0
Too long	1	2.0	7	14.0	8	8.0
*χ*^2^(2)=4.97; *p* = 0.083						
Fitting the footwear in terms of length –left foot						
Too short	16	32.0	12	24.0	28	28.0
Appropriate	33	66.0	31	62.0	64	64.0
Too long	1	2.0	7	14.0	8	8.0
*χ*^2^(2) = 5.13; *p* = 0.077						
Fitting the footwear in terms of width –right foot						
Too narrow	28	56.0	25	50.0	53	53.0
Appropriate	13	26.0	17	34.0	30	30.0
Too wide	9	18.0	8	16.0	17	17.0
*χ*^2^(2) = 0.76; *p* = 0.683						
Fitting the footwear in terms of width –left foot						
Too narrow	21	42.0	26	52.0	47	47.0
Appropriate	16	30.0	16	32.0	32	32.0
Too wide	13	26.0	8	16.0	21	21.0
*χ*^2^(2) = 1.72; *p* = 0.422						

**Notes.**

*n* number of subjects % percent of subjects *χ*^2^ value of the Chi-square test statistic *p* probability value

Table 5 provides the structural parameters of regression models, relating to a group of girls. Models defining the influence of such variables as length and width functional excess of indoor shoes on Clarke’s angle proved to be statistically significant for both the right foot (*F* = 10.08, and *p* < 0.001) and the left one (*F* = 8.28, and *p* = 0.001). The values of the coefficients R^2^ show that in the case of the right foot, the predictors explained a total of 30% of the variance of the dependent variable and in the case of the left one 26% of the variance of the dependent variable. While analyzing the influence of each predictor separately on the dependent variable it turned out that the size of the functional length excess has a statistically significant impact on Clarke’s angle of the right (r_p_ =  − 0.53, and *p* < 0.001) and left foot (r_p_ =  − 0.51, and *p* < 0.001). The value of the coefficient b of slope of the regression line points that decrease in the length excess by a unit will affect the growth of the Clarke’s angle by 1.39° for right foot and 1.45° for left foot.

**Table 5:** Regression models in girls.

**Excess of footwear [mm]**	***R***	***R*^2^**	**${R}_{\mathrm{adj}}^{2}$**	**F**	*p*	**b**	**r_p_**	*p*
**Clarke’s angle [^∘^] –right foot**								
**Length**	0.55	0.30	0.27	10.08	<0.001^*^	−1.39	−0.53	<0.001^*^
**Width**						−0.25	−0.16	0.260
**Clarke’s angle [^∘^] –left foot**								
**Length**	0.51	0.26	0.23	8.28	0.001^*^	−1.45	−0.51	<0.001^*^
**Width**						−0.03	−0.02	0.898
**Heel angle (*γ*) [^∘^] –right foot**								
**Length**	0.37	0.14	0.10	3.68	0.033^*^	−0.28	−0.32	0.025^*^
**Width**						0.13	0.21	0.139
**Heel angle (*γ*) [^∘^] –left foot**								
**Length**	0.20	0.04	0.00	1.01	0.372	−0.01	−0.01	0.936
**Width**						0.13	0.20	0.162
**Hallux valgus angle (*α*) [^∘^] –right foot**								
**Length**	0.05	0.00	−0.04	0.06	0.936	0.07	0.04	0.802
**Width**						−0.04	−0.04	0.787
**Hallux valgus angle (*α*) [^∘^] –left foot**								
**Length**	0.06	0.00	−0.04	0.07	0.927	0.05	0.02	0.881
**Width**						−0.06	−0.05	0.708
**The angle of the varus deformity of the Vth toe (*β*) [^∘^] –right foot**								
**Length**	0.23	0.05	0.01	1.36	0.264	−0.49	−0.16	0.272
**Width**						0.39	0.18	0.209
**The angle of the varus deformity of the Vth toe (*β*) [^∘^] –left foot**								
**Length**	0.30	0.09	0.05	2.45	0.097	−0.79	−0.29	0.051^*^
**Width**						−0.09	−0.06	0.669

**Notes.**

*R* coefficient of multiple correlation *R*^2^ coefficient of determination *R*_adj_^2^ adjusted *R*^2^ F value of the Fisher-Snedecor test statistic b coefficient of slope of the regression line *r*_*p*_ partial correlation *p* probability value

* *α* < 0.05.

The influence of prediction variables on the values of the heel angle (*γ*) proved to be statistically significant in case of the right foot (*F* = 3.68, and *p* = 0.033). Together these variables explained 14% of the heel angle variation. The values of the partial correlation coefficients showed that influence of the length excess of the indoor footwear on the heel angle was the only one to be statistically significant: *r*_p_ =  − 0.32, and *p* = 0.025. The value of the coefficient b for variables: the length excess of the indoor shoes and the right foot heel angle demonstrate that with the decline of the shoe length by a unit, the heel angle increases on average by 0.28°.

The data presented in Table 6 indicate that in boys regression models including 2 explanatory variables of Clarke’s angle variation turned out to be statistically significant for both the right (*F* = 17.86, and *p* < 0.001) and left foot (*F* = 13.89, and *p* < 0.001). Considering the right foot, the predictors allowed to explain a total of 43% of the dependent variable variance, and 37% of the dependent variable variance for the left foot. The values of the partial correlation coefficients showed statistically significant, negative effect of the length excess of the indoor footwear on Clarke’s angle of the right (r_p_ =  − 0.65, and *p* < 0.001) and left foot (r_p_ =  − 0.57, and *p* < 0.001). The value of the coefficient b points that the decrease in the length excess by a unit will affect the growth of the Clarke’s angle by 2.87° for right foot and 2.31° for left foot.

**Table 6:** Regression models in boys.

**Excess of footwear [mm]**	***R***	***R*^2^**	***R*^2^_adj_**	**F**	*p*	**b**	**r_p_**	*p*
**Clarke’s angle [^∘^] –right foot**								
**Length**	0.65	0.43	0.41	17.86	<0.001^*^	−2.87	−0.65	<0.001^*^
**Width**						−0.25	−0.16	0.260
**Clarke’s angle [^∘^] –left foot**								
**Length**	0.61	0.37	0.34	13.89	<0.001^*^	−2.31	−0.57	<0.001^*^
**Width**						−0.26	−0.11	0.448
**Heel angle (*γ*) [^∘^] –right foot**								
**Length**	0.35	0.12	0.08	3.22	0.048^*^	−0.29	−0.33	0.019^*^
**Width**						0.02	0.03	0.837
**Heel angle (*γ*) [^∘^] –left foot**								
**Length**	0.20	0.04	0.00	0.95	0.392	0.03	0.03	0.936
**Width**						0.10	0.18	0.225
**Hallux valgus angle (*α*) [^∘^] –right foot**								
**Length**	0.34	0.12	0.08	3.05	0.056	0.48	0.33	0.020^*^
**Width**						0.00	0.00	0.984
**Hallux valgus angle (*α*) [^∘^] –left foot**								
**Length**	0.39	0.15	0.12	4.25	0.020^*^	0.66	0.36	0.011^*^
**Width**						0.06	0.06	0.699
**The angle of the varus deformity of the Vth toe (*β*) [^∘^] –right foot**								
**Length**	0.27	0.08	0.03	1.90	0.159	0.05	0.03	0.847
**Width**						−0.39	−0.27	0.058
**The angle of the varus deformity of the Vth toe (*β*) [^∘^] –left foot**								
**Length**	0.15	0.02	−0.02	0.57	0.568	0.32	0.13	0.365
**Width**						−0.20	−0.12	0.424

**Notes.**

*R* coefficient of multiple correlation *R*^2^ coefficient of determination *R*_adj_^2^ adjusted *R*^2^ F value of the Fisher-Snedecor test statistic b coefficient of slope of the regression line *r*_*p*_ partial correlation *p* probability value

* *α* < 0.05.

Regression model including 2 explanatory variables of heel angle (*γ*) variation was statistically significant only in the case of the right lower limb: *F* = 3.22, and *p* = 0.048. These variables accounted for a total of 12% of the variance of the dependent variable. The values of the partial correlation coefficients showed a statistically significant negative effect of the length excess of the indoor footwear on the heel angle: *r*_p_ =  − 0.33, and *p* = 0.019. The value of the coefficient b points that decrease of the length of a shoe by a unit results in rise of heel angle by an average of 0.29°.

The overall impact of predictors of indoor footwear on the hallux valgus angle (*α*) value was statistically significant only for the left foot: *F* = 4.25, and *p* = 0.020 (*R*^2^ = 0.15), although the values of the partial correlation coefficients showed that the size of the length excess has a statistically significant impact on hallux valgus angle of the right (*r*_p_ = 0.33, and *p* = 0.020) and left foot (*r*_p_ = 0.36, and *p* = 0.011). The values of the coefficients b points that the increase in the length excess by a unit will affect the growth of the hallux valgus angle by 0.48° for right foot, and 0.66° for left foot.

## Discussion

The measure of the degree of fitting the footwear to the foot is the functional excess, which on the one hand is a reserve for growing feet, and on the other hand protects the free space in the footwear needed to extend the longitudinal arch (the so-called apparent increase in the length of the foot) during movement or load. Our results show that girls’ indoor footwear is characterized by a smaller functional excess of length. These data are consistent with the results of Delgado-Abellán et al. (2014), who examinated children aged 6–12 years attending primary schools in Spain. The authors compared the size marked on the child’s footwear with the length of the foot. Comparison between the real size of the shoe and the size estimated from the foot length showed that girls wore shoes that were smaller than what they should have worn. The authors in the same study showed that in this age category the average annual increase in foot length is smaller in girls than in boys and amounts to 3.5% and 5%, respectively.

In our study, the size of the functional width excess did not differentiate the examined girls and boys. Due to the fact that there are no reports in the literature on the intergender comparisons of footwear fit in terms of width, these results are difficult to compare with the results of other authors.

Wearing properly fitted footwear, especially in terms of length and width, can prevent abnormalities resulting from reduced mobility and free deformation of the foot arches. Our study has shown that the right and left feet of 7-year-old girls and boys are characterized by the symmetry of length and width. These data are consistent with the reports of Vrdoljak & Tiljak (2017) based on study results of children living in the Republic of Croatia. The symmetry of length and width creates the comfort of buying footwear that is equally fit for both feet. Pavlackova et al. (2015) highlighted that foot length is a crucial dimension in selecting the most appropriate size of footwear. According to Zhang & Wang (2017) most children, especially in the early stages of ontogenesis, wear shoes that are too short. In our study, as many as 40% of girls and boys had shoes that were incorrectly fitted in length, which were often too short, while shoes that were incorrectly fitted in width were worn by as many as 74% of girls and 66% of boys (most of them used shoes that were too short), and the gender influence the obtained results González Elena & Córdoba-Fernández (2019) based on a study from southern Spain, found that 72% of the surveyed children aged 3-12 years old, regardless of gender, wore shoes that were too short, and 67% wore shoes that were too narrow. Yurt, Sener & Yakut (2014) and Delgado-Abellán et al. (2014) analyzed the issues of footwear fit in terms of width. The authors indicated that most footwear designs do not take into account the need to choose different widths for one length size. González Elena & Córdoba-Fernández (2019) concluded that when designing ergonomic footwear for schoolchildren, not only the different digital formulae must be taken into account, but also the position and orientation of the forefoot inside the shoe and its interaction with the shoe tip.

It is worth emphasizing, user subjective feelings about footwear comfort are significantly important when selecting and wearing footwear (Zhang & Wang, 2017). Footwear that is too tight puts pressure on the tissue of the foot, making it uncomfortable. Conversely, when the footwear is too loose, the inside of the foot slips while walking, which increases physical exertion and potentially causes a frictional injury to the foot’s soft tissues (Pavlackova et al., 2015). In our study, despite the lack of specific data on the fit of the footwear, we obtained favourable results regarding the comfort of its use. Such a discrepancy in the objective and subjective assessment of footwear fit may result from the fact that the foot adjusts to poorly selected shoes, proprioception changes, and therefore its incorrect positioning in the shoe initially does not cause discomfort, but over time it affects the formation or deepening of deformation and even micro-damage to its delicate structures. The discrepancies in the results obtained in objective and subjective examinations question the validity of using the visual-analogue scale in 7-year-old children, but these issues require separate scientific studies. An interesting issue is the influence of the size of the functional excess of footwear on the shape of the foot. Our study has shown that in girls and boys, the length of the footwear has an influence on the longitudinal arch of the right and left foot. Regression analysis has shown that reducing the shoe length by one unit increases the value of the Clarke’s angle. These results allow the conclusion that wearing shoes that are too short causes excessive arching of the foot. The lack of free space in the footwear makes it difficult to extend the foot during locomotion, contributes to the restriction of the movements of its individual components, and as a consequence may cause contracture of the plantar fascia and short flexor muscles of the foot. We also observed the effect of wearing too short shoes on the increase in the value of the heel angle of the right foot, which may be caused by a change in pressure distribution.

In girls, the length and width of indoor shoes do not significantly affect the position of the hallux and fifth toe, while in boys, the length of the shoes shows associations with the right and left hallux valgus angle. This suggests that due to the deformity of the first metatarsophalangeal joint, the boys with hallux valgus require footwear which is wider and therefore their shoes need to be bigger in size. These results are inconsistent with the data reported by Klein et al. (2009) who found increasing of hallux valgus in preschool children wearing too short shoes.

Our findings gives sufficient grounds to believe that wearing of the improperly selected footwear becomes an independent risk factor for accelerating a number of unfavorable changes in the foot structure. In view of the sheer scale of the problem, frequently caused by blind pursuit of fashion trends and/or negligence, it may be assumed that steadily more children struggle with feet problems. Specific data yielded by our research may well serve as a source of reliable information to both the footwear manufacturers, as well as the children parents. The authors subscribe to the view that a key component in raising awareness about this problem would consist in developing comprehensive instruction schemes promoting overall footwear safety.

### Limitations and future research

The scope of this cross-sectional study has been narrowed down to one area and one age category, which may be a limitation of the study. Future research could be planned in the form of a controlled experiment, extended to other regions of the country and also covering other age categories. Such a procedure will allow for a more detailed understanding of the cause and effect relationships regarding the impact of wearing specific types of footwear on the structure of the foot.

## Conclusions

1. In terms of length, width, longitudinal arch, position of the hallux, the right and left feet of the examined children are characterized by symmetry. In girls, there were no differences in the transverse arches of the right and left feet, while in boys, the right feet were transversely flatter than the left ones. In both sexes, the angle of the varus deformity of the Vth toe of the right foot was greater than that of the left toe.
2. About 40% of girls and boys had shoes that were incorrectly fitted in length, while as many as 74% of girls and 66% of boys wore shoes that were incorrectly fitted in width. The frequency of wearing improperly fitted shoes did not depend on gender.
3. Girls’ indoor footwear was characterized by a smaller functional length excess. The size of the functional excess of the width did not differentiate the examined girls and boys.
4. In both sexes, the length of the footwear influenced the longitudinal arch of the right and left foot and the transverse arch of the right foot. In boys, the length of the shoes shows associations with the right and left hallux valgus angle. This suggests that due to the deformity of the first metatarsophalangeal joint, the boys with hallux valgus require footwear which is wider and therefore their shoes need to be bigger in size.

##  Supplemental Information

10.7717/peerj.11277/supp-1 Supplemental Information 1 Raw data applied for data analyses and preparation for Tables 1–6

10.7717/peerj.11277/supp-2 Supplemental Information 2 Numerical and categorical data

## Additional Information and Declarations

### Data Availability

The following information was supplied regarding data availability: The raw measurements are available in the Supplemental Files.
